# Molecular analysis of *Sarcoptes scabiei* infecting wild and domestic South American camelids in Argentina

**DOI:** 10.1017/S0031182025000344

**Published:** 2025-04

**Authors:** Melina Anello, Fabiana Sosa, Hebe Ferreyra, Rebeca Lobo Allende, Mariana Mastromatey, Marcela Uhart, Sandra Romero, Mónica Florin-Christensen, Barbara Moroni, Anna Rita Molinar, Luca Rossi, Florencia Di Rocco

**Affiliations:** 1 Instituto Multidisciplinario de Biología Celular CONICET-UNLP-CIC, La Plata, BA, Argentina; 2 Instituto de Investigación y Desarrollo Tecnológico para la Agricultura Familiar, Región NOA, INTA, Argentina; 3 Consejo Nacional de Investigaciones Científicas Y Técnicas (CONICET), Argentina; 4 Dirección Nacional de Conservación, Delegación Regional Centro de la Administración de Parques Nacionales, Argentina; 5 Universidad Nacional de Villa María, Córdoba, Argentina; 6 Universidad Nacional de Chilecito, La Rioja, Argentina; 7 Latin America Program, Karen C. Drayer Wildlife Health Center, School of Veterinary Medicine, University of California, Davis, USA; 8 Instituto de Patobiología Veterinaria, INTA-CONICET, Centro de Investigaciones en Ciencias Veterinarias y Agronómicas, INTA, Argentina; 9 Istituto Zooprofilattico Sperimentale di Piemonte, Liguria e Valle d’Aosta, Torino, Italy;; 10 Departamento de Ciencias Veterinarias, Universidad de Torino, Grugliasco, Italy

**Keywords:** genetic composition, guanaco, high-altitude livestock farming, llama, sarcoptic mange, vicuña, wildlife conservation

## Abstract

Sarcoptic mange, caused by the *Sarcoptes scabiei* mite, is a highly transmissible skin condition affecting many mammalian species worldwide. South American camelids (SAC) have the highest reported prevalence of mange in South America, causing economic losses and posing a conservation threat to wild SAC. This study investigated mite diversity in SAC in Argentina and assessed relationships between known outbreak areas. Distinct epidemiologic scenarios were explored: the San Juan-La Rioja region, where a mange outbreak decimated wild SAC populations, and the Puna region of Jujuy, where domestic and wild SAC coexist and infections often occur. The mitochondrial gene *cox1* and ten microsatellites were analysed from mites collected in five sampling events in Jujuy and four in San Juan-La Rioja between 2017 and 2023. A single *cox1* haplotype was observed regardless of mite origin or host species. Comparison with partial *cox1* sequences from other camelids worldwide showed little variation. Microsatellite markers revealed lower diversity in mites from San Juan-La Rioja compared to Jujuy. A single strain common to vicuñas and guanacos was identified in San Juan-La Rioja, while three strains were detected in Jujuy affecting vicuñas and/or domestic llamas. Some mites from Jujuy exhibited mixed genetic composition between the two regions, and results confirmed that domestic and wild SAC shared mite strains. This study enhances understanding of sarcoptic mange transmission among SAC species, contributing to vicuña and guanaco conservation and high-altitude livestock farming. Additionally, these findings provide support for the development of intersectoral management strategies to address this significant threat.

## Graphical abstract

**Figure:**
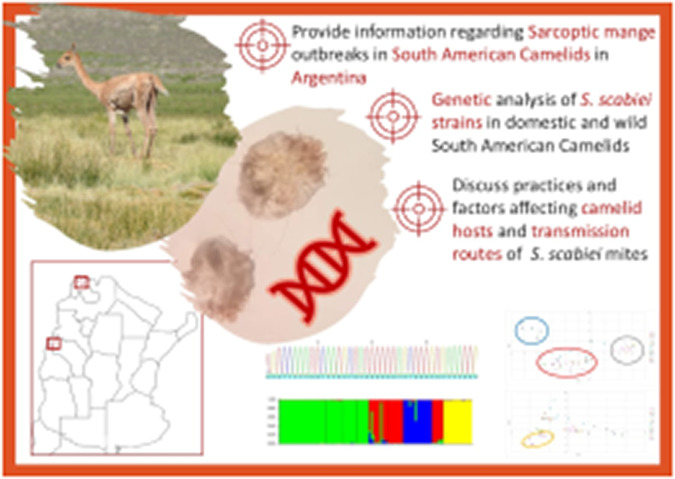

## Introduction

Sarcoptic mange, caused by the mite *Sarcoptes scabiei*, is a well-known highly contagious skin disease. It has been reported in more than 100 mammalian species around the world, and cases continue to emerge in new hosts and geographical locations (Astorga et al., 2018). Epidemiologic scenarios in wildlife may range from severe outbreaks with the risk of local extinction of susceptible host nuclei to long-term endemicity, where the disease has a relatively low prevalence and has low demographic impact (Browne et al., 2022). Although the first scenario is less common, catastrophic population declines have been reported in Northern chamois *(Rupicapra rupicapra)* and Alpine ibex *(Capra ibex)* (Rossi et al., 1995, 2007), Iberian ibex *(Capra pirenaica)* (León-Vizcaíno et al., 1999), red foxes *(Vulpes vulpes)* (Soulsbury et al., 2007), San Joaquin kit foxes *(Vulpes macrotis)* (Cypher et al., 2017), and bare-nosed wombats *(Vombatus ursinus)* (Martin et al., 2018). Endemicity has been documented for those same species as well as for others that have never experienced severe outbreaks (Gortázar et al., 1998; Martin et al., 1998; Soulsbury et al., 2007; Rossi et al., 2019; Carver et al., 2021).

In South America, reports of mange in wildlife have increased considerably in recent decades, affecting many species, including South American camelids (SAC). This taxonomic group is the most affected by sarcoptic mange, with reports from Argentina, Bolivia, Chile, and Peru (Beltrán-Saavedra et al., 2011; Arzamendia et al., 2012; Gomez-Puerta et al., 2013, 2022; López Jiménez, 2018; Montecino-Latorre et al., 2020; Acebes et al., 2022; Ferreyra et al., 2022; Sosa et al., 2022, 2025). Both domestic, llama (*Lama glama*) and alpaca (*Vicugna pacos*), and wild camelid species, vicuña (*Vicugna vicugna*) and guanaco (*Lama guanicoe*), are affected. However, there is a major and growing concern related to conservation of the latter, especially after the recent devastating outbreak in San Guillermo National Park (SG), San Juan province, Argentina (Donadio and Perrig, 2017). Currently, guanacos and vicuñas are ecologically locally extinct in SG, with evidence showing that the outbreak has affected the whole ecosystem by altering trophic interactions (Monk et al., 2022). Ferreyra et al. (2022) studied the dynamics of the mange outbreak and its effects on SG wild camelid populations. They hypothesized that the outbreak originated from the government-led introduction of domestic llamas from Jujuy province in northern Argentina to neighbouring areas of SG. It is also alleged that mange may have spread from SG to other off-park wild camelid populations, mainly by guanacos through their migratory movements (Ferreyra et al., 2022), since cases of mangy vicuñas have since been reported in the neighbouring provinces of La Rioja (Castillo Sánchez, 2018) and Catamarca (R. Lobo and M. Mastromatey personal observation).

**Figure 1.**
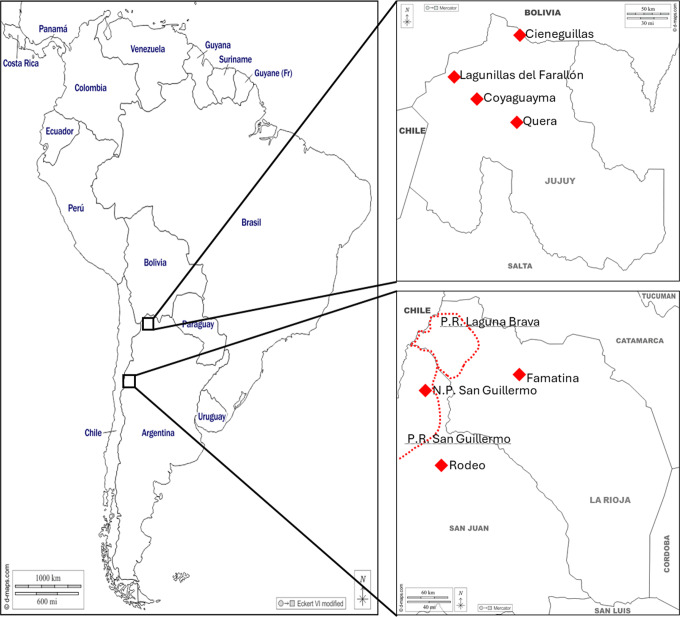
Geographic locations of the sampling sites. Left: map of South America showing the regions where samples were obtained for this study (black-bordered squares). Right top: close-up map of jujuy province. Right-bottom: close-up map of San Juan and La Rioja provinces. The sampling sites are denoted by red rhombuses, and the boundaries of the provincial reserves (P.R.), where the samples were collected, are indicated by dotted red lines. P.R. names are underlined. All maps were obtained from https://d-maps.Com and modified accordingly for this study.

In the Puna region of Jujuy Province, domestic llamas coexist with wild vicuñas (and a very small guanaco population), and it is common to find other livestock, such as sheep, grazing together with vicuñas as well (Vilá, 2015). Llamas are bred for their meat and fibre, an activity of high socio-economic relevance in the province, where the largest Argentine population of llamas is found (Marin et al., 2022). Furthermore, in Jujuy, vicuñas are managed for live shearing through annual captures by Andean communities, following the ancestral practice of chaku (Lichtenstein and Vilá, (2003); Vilá and Arzamendia, 2022), representing a sustainable system that generates income from valuable vicuña fibre for local communities (Quispe et al., 2009). Chakus are also an opportunity for animal health surveillance and mange detection, although treating wild populations is prohibited. To the best of our knowledge, the first mange report of vicuñas from the Puna region of Jujuy was during captures carried out in 2003–2005 in Cieneguillas (CN; see Figure 1) with a low prevalence (Arzamendia et al., 2012). In 2014, two other outbreaks of sarcoptic mange were subsequently reported nearby in the Puna region in a llama herd and in a group of vicuñas kept in captivity (Aráoz et al., 2016). During 2018 and 2019, mange was observed during vicuña chakus in Lagunillas del Farallón (LF; see Figure 1), with its occurrence increasing over the one-year period (Mir et al., 2019; Sosa et al., 2022). Additionally, in 2019 a few affected animals were observed in Coyaguayma (approximately 40 km from LF; Figure 1) and, a single mangy vicuña was detected in Quera (approximately 75 km away from LF; Figure 1) (Sosa et al., 2022). The increase in mange cases in LF is alarming, and the proximity with the other sites suggests that the disease might have spread to neighbouring populations.

Even though sarcoptic mange is common in SAC and both endemic and epidemic scenarios have been described, most of the available descriptive epidemiologic information is found in the ‘grey literature’ (Acebes et al., 2022; Moreno et al., 2024). Moreover, few genetic studies have focused on the mites that cause the disease in SAC. Gomez Puerta *et al*. (2022) used mitochondrial (cytochrome C oxidase subunit I, *cox1*) and nuclear (internal transcribed spacer 2, ITS2) genetic markers to characterize mites from vicuñas in the southern Peruvian Andes and reported that only *cox1* presented polymorphisms associated with the host species. Sosa et al. (2022; 2024; 2025 also used *cox1* to detect the etiological agent from the Jujuy-2019 chakus and form of llamas of Jujuy province, whereas Ferreyra et al. (2022) employed microsatellite markers to determine that guanacos and vicuñas from SG were infected by the same variant of *S. scabiei*. In other species, molecular markers have enabled the identification of mite transmission pathways, revealing different inter- and intraspecific transmission patterns (Rossi et al., 2023). For example, Rasero et al. (2010) used microsatellite markers to analyse mites isolated from 10 wild species in Europe and identified three genetically different groups, namely, herbivorous, carnivorous and omnivorous hosts. In another study, Andriantsoanirina et al. (2015) reported that *S. scabiei* affecting humans, is distributed in three well-differentiated groups and that there is limited genetic flow between mites from one human group and mites from animal species.

The aim of this study was to explore mite diversity in SAC in Argentina and assessed the relationships between known outbreak areas, thus ultimately contributing to vicuña and guanaco conservation in the Southern Andes. In particular, the objectives were to (1) provide further information regarding the SG outbreak, its alleged expansion to neighbouring sites and its putative origin; (2) analyse mite variants occurring in domestic llamas and wild vicuñas from Jujuy; and (3) discuss practices and factors affecting the investigated camelid hosts and the transmission routes of the respective mites.

## Materials and methods

### Samples, mite isolation and DNA extraction

The samples consisted of skin scrapings from living or dead camelids with mange-compatible signs (n=38), such as intense scratching, difficulty walking, thickening, rusty or cracked skin, and alopecia or ruffled or detached fleece fibres (Ferreyra et al., 2022). Scrapings were collected from llamas and vicuñas from the Puna region of Jujuy and from guanacos and vicuñas from the provinces of San Juan and La Rioja. Figure 1 shows the geographic locations of the sampling sites. The samples were grouped according to geographic location and sampling year. Table 1 includes more detailed information and an abbreviation for each group that is used in the rest of this study. The samples from SG1 (San Guillermo National Park) correspond to those from the study of Ferreyra et al. (2022); the vicuña samples from LF_V_ (Lagunillas del Farallón- vicuñas), QU (Quera), and CY (Coyaguayma) correspond to the cases described by Sosa et al. (2022); and the llama samples from LF_L_ (Lagunillas del Farallón- llamas) correspond to the cases described by Sosa et al. (2025).

**Table 1.** Samples used in this study. An abbreviation for each group of samples is included as well as geographical origin, sampling year, host species sampled, and number of mites analysed for microsatellite markers. LF refers to ‘Lagunillas del Farallón,’ but since vicuñas and llamas were sampled in that region at differed times, they are referred to as LF_V_ and LF_L_, respectively.

Abbreviation	Geographical origin	Sampling year	Host species sampled (n)	Mites analysed (microsatellites)
**SG1**	San Guillermo National Park, San Juan	2017–2018	*Lama guanicoe* (3) *Vicugna vicugna* (6)	24
**SG2**	San Guillermo Provincial Reserve and Rodeo surroundings, San Juan	2022–2023	*Lama guanicoe* (1) *Vicugna vicugna* (2)	9
**LB1**	Laguna Brava Provincial Reserve, La Rioja	2019	*Lama guanicoe* (2) *Vicugna vicugna* (2)	8
**LB2**	Laguna Brava Provincial Reserve and Famatina surroundings, La Rioja	2023	*Lama guanicoe* (1) *Vicugna vicugna* (1)	6
**CY**	Coyaguayma, Jujuy	2021–2023	*Vicugna vicugna* (3)	10
**QU**	Quera, Jujuy	2019–2021	*Vicugna vicugna* (3)	8
**LF_V_**	Lagunillas del Farallón, Jujuy	2019–2021	*Vicugna vicugna* (5)	15
**LF_L_**	Lagunillas del Farallón, Jujuy	2021–2022	*Lama glama* (2)	6
**CN**	Cieneguillas, Jujuy	2018	*Lama glama* (7)	15
	**Total**		38	101

The presence of *S. scabiei* in each skin scrap sample was confirmed via microscopic observation 3–5 days after collection. Taxonomic identification based on morphological characteristics was carried out according to Arlian and Morgan (2017). Mites were recovered and placed in 70% ethanol for subsequent DNA extraction. DNA was extracted from individual mites following the HotSHOT Plus ThermalSHOCK technique (Alasaad et al., 2008).

### Molecular markers

For this study, two types of molecular markers were used: the mitochondrial gene *cox1* and a set of 10 mite microsatellites.

A pair of primers was designed via the PrimerBLAST tool (Ye et al., 2012) on the basis of the complete mitochondrial genome sequence of *S. scabiei* isolated from humans (accession number: LN874268.1) to amplify the complete mitochondrial *cox1* gene with its flanking regions (1959 bp) (Table S1). Once the complete *cox1* sequence was obtained, due to the low amount of DNA recovered per mite, PCR amplification of two consecutive fragments of 619 pb and 573 pb was performed for further sequencing (Table S1). A total of 27 mites were sequenced for *cox1* (details of the sequenced samples can be found in Table S2). PCRs were carried out in a 25 μl mixture containing 1X PCR buffer (PB-L, Pegasus), 2.5 mM MgCl2, 0.2 mM dNTPs, 1 U Taq DNA polymerase (PB-L, Pegasus), 0.5 μM of each primer, and 5‒6 μl of extracted DNA. The cycling profile consisted of an initial denaturation step at 94°C for 3 min; 30 cycles of 40 s at 94°C, 90 s at 54/62°C and 40 s at 72°C; and a 10-min final extension at 72°C. The PCR products were checked via electrophoresis in 1% agarose gels stained with Gel Red (Biotium) and with the GeneRuler 1 kb Plus DNA Ladder (Thermo Scientific). Amplicons of the expected size were purified by polyethylene glycol (PEG) precipitation and then sequenced at Macrogen, Inc.

A set of 10 mite microsatellite markers (SARMS 33, 34, 35, 36, 37, 38, 40, 41, 44 and 45) (Rasero et al., 2010) was amplified in a multiplex PCR as described in Moroni et al. (2021) for the 101 mites isolated (details of mite samples analysed are shown in Table 1). The PCR products were separated by electrophoresis using SeqStudio Genetic Analyzer. Microsatellite fragment sizes and alleles were assigned with GeneMapperTM software.

### Data analysis

*cox1* sequences were compared with those deposited in GenBank and aligned with the BLAST tool (https://blast.ncbi.nlm.nih.gov/Blast.cgi).

Standard genetic diversity parameters for microsatellite data, such as observed (Ho) and expected (He) heterozygosities, Hardy–Weinberg equilibrium, number of alleles (N_A_) and number of private alleles (A_P_), were calculated using GenAlEx v6.5 (Peakall and Smouse, 2012). The FSTAT 2.9.4 program was used to estimate allelic richness (A_R_).

To test for genetic differentiation among mites from different locations, we calculated Fst coefficients using GenAlEx v6.5, as well as analysis of molecular variance (AMOVA). We also performed a principal component analysis (PCA) via the R package ‘adgenet’ and a principal coordinate analysis, also known as metric multidimensional scaling (MDS), as implemented in GenAlEx v6.5. Both the PCA and MDS methods are used for dimensionality reduction and data visualization. While PCA focuses on capturing maximum variance in the data to represent the population structure on the basis of genetic correlations among individuals, MDS detects meaningful underlying dimensions that explain the observed genetic distance among individuals (Wang et al., 2009).

Additionally, a Bayesian clustering analysis was performed with the STRUCTURE program (Pritchard et al., 2000) using a burn-in period of 100,000 followed by the same number of MCMC replications. An admixture ancestry model with sampling location as prior information was selected. The number of populations (K) varied from 1 to 9, and 10 iterations for each K were performed. The assignment of the most likely K was made taking into account the maximum value of ln [Pr (X/K)] and the individual assignment values (q values) (Evanno et al., 2005).

## Results

The morphologic characteristics of the mites were consistent with *Sarcoptes scabiei*. They exhibited a globular idiosome, thick gnathostome, dorsal spines, and four pairs of leg with suction cups on the first two pairs (Fig. S1).

All the samples analysed for *cox1* presented the same haplotype, regardless of their origin or host species. The *cox1* sequences obtained were uploaded to GenBank under accession numbers PQ137498-PQ137524 (Table S2). The sequence was compared with other *cox1* sequences from *S. scabiei* available in GenBank to date. However, most of those available represent partial sequences, and only 16 encompass complete *cox1* sequences. The comparison revealed that the *cox1* haplotype isolated from mites from Argentinian SAC was not previously registered for *S. scabiei*. This haplotype presented an identity of 100% with a 421 bp fragment of *S. scabiei* isolated from vicuñas from the southern Peruvian Andes (GenBank accession number MZ569487.1) (Gomez-Puerta et al., 2022) (Fig. S2A) and an identity of 99% with a second haplotype described in the same study (GenBank accession number MZ569453.1) (Fig. S2B). Furthermore, it also showed 99% identity with a 484 bp fragment isolated from camels (*Camelus dromedarius*) in Iraq (GenBank accession number OK510218.1) (Al-Hasnawy et al., 2022) (Fig. S2C).

With respect to microsatellite markers, a total of 40 alleles were detected for the 101 mites analysed, ranging from one allele at SARMS-36 to seven at SARMS-34. At the San Juan and La Rioja sites, where mites from both guanaco and vicuña were collected, the same genotype was obtained regardless of the species. Thus, Table 2 shows the combined genetic diversity parameters for both species per sampling site. Significant deviations from Hardy‒Weinberg equilibrium were observed for at least one locus at all the sites, except for LB1 (Laguna Brava Provincial Reserve), LB2 (Laguna Brava Provincial Reserve and Famatina surroundings) and LF_L_, which had the highest proportion of monomorphic loci (Table 2, Fig. S3). The observed and expected heterozygosity across all the loci and sites were Ho=0.039±0.008 and He=0.095±0.016 (mean±SE). In general, mites from Jujuy Province presented higher diversity values than did mites from San Juan-La Rioja, particularly CY, which presented the highest number of alleles and the highest percentage of polymorphic loci (Table 2). Additionally, CY had the highest allelic richness, which is a measure of the number of alleles corrected by sample size. Private alleles were detected within all the sites except LB1 and LF_L_, which was probably due to a relatively small sample size.

**Table 2.** Genetic diversity parameters of 10 microsatellite loci from *S. scabiei* mites from different SAC species and geographical origins

Geographical origin		N	N_A_	A_R_	A_P_	%P	Ho ±(SE)	He ±(SE)
**San Juan- La Rioja**	**SG1**	24	14	1.135	1	40%	0.026 (0.018)	0.047 (0.021)
	**SG2**	9	10	1.185	1	30%	0.033 (0.024)	0.075 (0.046)
	**LB1**	8	11	1.038	0	10%	0.013 (0.013)	0.012 (0.012)
	**LB2**	6	12	1.127	2	20%	0.050 (0.036)	0.043 (0.030)
**Jujuy**	**CY**	10	22	1.775	2	90%	0.113 (0.040)	0.284 (0.057)
	**QU**	8	12	1.125	3	20%	0.000 (0.000)	0.044 (0.029)
	**LF_V_**	15	18	1.304	3	60%	0.040 (0.018)	0.106 (0.041)
	**LF_L_**	6	12	1.149	0	20%	0.033 (0.022)	0.064 (0.049)
	**CN**	15	17	1.425	4	60%	0.040 (0.018)	0.179 (0.067)

Table Abbreviations. N: number of mites analysed; N_A_: total allele number; A_R_: allelic richness; A_P_: number of private alleles; %P: percentage of polymorphic loci; Ho: observed heterozygosity; He: expected heterozygosity; SE: standard error.

**Figure 2.**
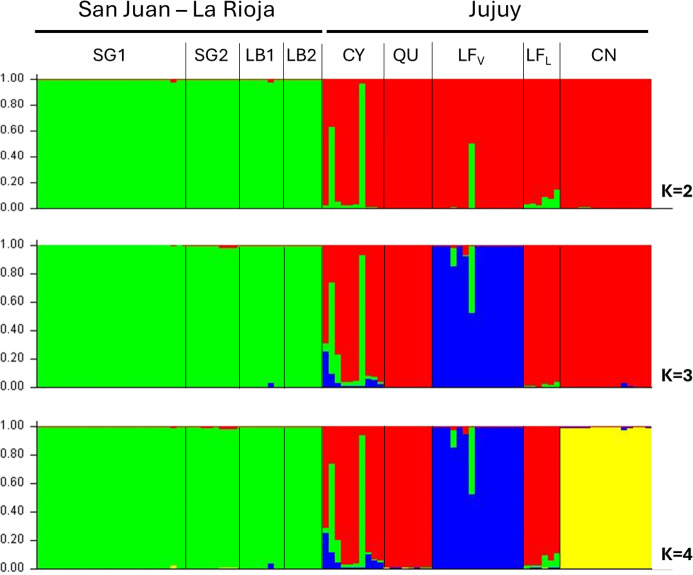
Structure plot for K clusters 2, 3, and 4. Each vertical line represents an individual mite. Geographic origins are listed above the plots with sample site abbreviations as in Table 1 and are separated by thin black lines. San Juan-La Rioja sites include mites from both host species, vicuñas and guanacos.

According to the AMOVA results, the highest variation was found between populations (71%) rather than between individual hosts (21%) or within individual hosts (7%) (Fig. S4), supporting origin-based differentiation rather than species-based differentiation. Similarly, Bayesian cluster analysis with the software STRUCTURE for K=2 grouped mites from San Juan-La Rioja into one cluster and mites from Jujuy into another (Figure 2). It can also be observed from the structural results that some mites from CY and one from LF_V_ presented a genetic composition with mixed components between the San Juan-La Rioja cluster and the Jujuy cluster. Notably, the same animals harbouring admixed mites were also infested by nonadmixed mites and were graphed next to each other in the structure plot in Figure 2. For K=3, the first cluster included all mites from San Juan-La Rioja, and the second cluster consisted of mites from Jujuy sites, except for LF_V_ which formed the third cluster (Figure 2). For K=4, all the mites from the CN llamas were separated from the rest, forming a third cluster within Jujuy Province (Figure 2). Using the method of Evanno *et al*. (Evanno et al., 2005), the number of clusters that best fit the data was determined to be 4 (Fig. S5).

**Figure 3.**
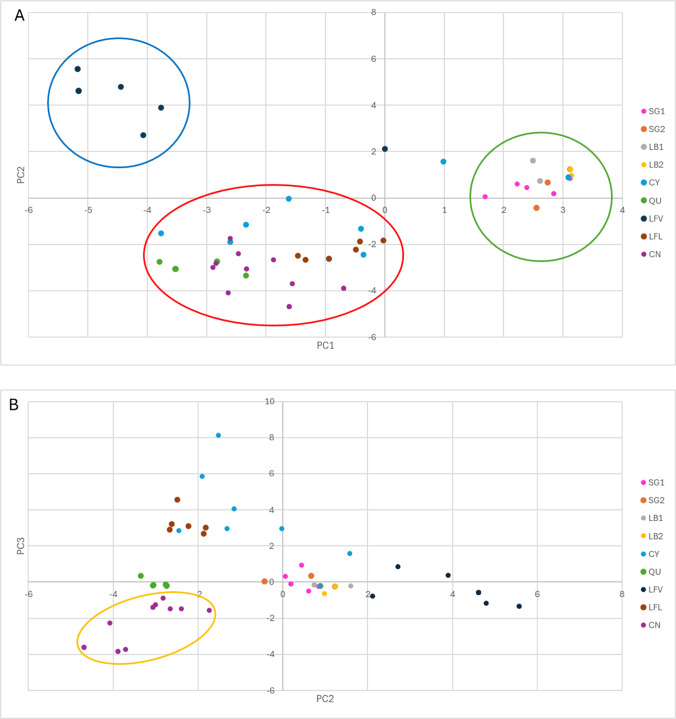
Principal component analysis (PCA) scatterplots for components 1 vs. 2 (A) and 2 vs. 3 (B). Each colour-coded dot represents a single mite from the corresponding sampling site, as indicated by the reference panel at the right of the plots. The main groups are encircled with similar colours to the structure plot for K=4: Blue corresponds to LF_V_; green encircles SG1, SG2, LB1 and LB2, the San Juan–La Rioja cluster; red groups Jujuy samples from CN, LF_L_, QU and CY; and yellow in the graph of principal components 2 vs. 3 indicates the differentiation of CN from the Jujuy cluster.

The PCA and MDS results were concordant with the Bayesian clustering results (Figures 3 and S6). The first two components in the PCA accounted for 16.05% of the total variance, and the scatterplot displayed the same three groups that were obtained for K=3 (Figure 3A). This plot also shows that there are samples from CY and LF_V_ that do not fully group with the remaining mites from their respective sites and are rather in between groups. This analysis also supported the further differentiation of CN from the Jujuy cluster when the third principal component was considered (Figure 3B).

The differentiation pattern was also clear according to the Fst results. The mean Fst value for the whole dataset was 0.707, and Table 3 shows pairwise comparisons. The most closely related mites were found in San Juan-La Rioja (Fst 0.033–0.089), and the greatest differences were between San Juan-La Rioja and QU (0.887–0.827) and San Juan-La Rioja and LF_V_ (0.842–0.713). Mites from CY presented intermediate Fst values compared with those of the other sites, as did LF_L_ and CN.

**Table 3.** Fst values for sampling site pairs estimated from ten microsatellite markers analysed from *S. scabiei* mites from different SAC species and geographical origins

	SG1	SG2	LB1	LB2	CY	QU	LF_V_	LF_L_	CN		
**SG1**	0.000										
**SG2**	0.048	0.000									0.000–0.100
**LB1**	0.033	0.080	0.000								0.100–0.400
**LB2**	0.045	0.089	0.056	0.000							0400–0.700
**CY**	0.358	0.333	0.393	0.387	0.000						0.700–1
**QU**	0.881	0.833	0.827	0.887	0.359	0.000					
**LF_V_**	0.713	0.745	0.842	0.807	0.458	0.685	0.000				
**LF_L_**	0.585	0.541	0.739	0.574	0.084	0.648	0.750	0.000			
**CN**	0.537	0.508	0.583	0.576	0.290	0.421	0.662	0.404	0.000		

Fst pairwise values are highlighted with an arbitrary scale of colour intensity to denote levels of differentiation, as indicated by the reference scale at the right of the table.

## Discussion

A molecular study of the mites that cause sarcoptic mange in SAC in Argentina is presented. A major result is the first description of S*. scabiei* genetic clusters (or ‘strains’) affecting both wild and domestic camelids. Moreover, strain distributions differ between regions and explored epidemiologic scenarios, involving only wild or mixed wild and domestic SAC. This study provides a baseline for improving the understanding of sarcoptic mange transmission among camelid species in South America, and provides relevant information for the management of this serious economic and conservation threat from an intersectoral perspective.

On the basis of the *cox1* genetic marker results, all analysed samples presented the same haplotype, although other haplotypes could also be present since not all the samples collected were sequenced for this marker. Nonetheless, the complete *cox1* haplotype appears to be a new sequence exclusive to SAC. The little variation of *cox1* partial sequences found among taxa support a host‒parasite adaptation hypothesis, at least at the host family level, as proposed by similar studies dealing with *S. scabiei* in other mammalian groups (Fraser et al., 2017; Rossi et al., 2023). Nevertheless, genetic studies on *S. scabiei* from other wild and domestic hosts that share habitats with SAC are necessary to confirm *cox1* exclusivity for the latter host group and to perform the necessary phylogenetic comparisons. An attempt was made to obtain *Sarcoptes* mites from other hosts, but no mange cases were identified during the study period. Unfortunately, sarcoptic mange is not a mandatory reportable disease in Argentina. Consequently, historical records of affected hosts and regions are unavailable, and many cases, especially in domestic species, go unnoticed. Moreover, some alleged mange cases in domestic animals are difficult to confirm, since treatment is often applied before or regardless of proper clinical examination (F. Sosa personal observation). In SG, other ongoing investigations are monitoring wildlife, and mange has not been registered in any other species in addition to vicuñas and guanacos, which may provide further evidence supporting a camelid-specific host‒parasite relationship.

The genetic diversity of mites measured by microsatellite markers was similar to that reported in studies in a range of different hosts infected by *S. scabiei* (Rasero et al., 2010; Rudd et al., 2020; Moroni et al., 2021). In general, we observed a low proportion of heterozygous mites (Ho: 0.039–0.113), even if deviation from HW equilibrium could indicate the presence of some null alleles, eventually leading to an underestimation of diversity. Low variation among individuals is expected when a few genotypes are spreading rapidly (Oura et al., 2005; Drees et al., 2017). Similarly, we observed that mites from San Juan-La Rioja, where the severe outbreak occurred, presented lower diversity values than those from Jujuy.

With respect to the Puna region of Jujuy, microsatellite analyses revealed three different genetic variants or strains affecting vicuñas and/or llamas. Interestingly, this clustering is neither strictly host nor site specific (Figures 2 and 3). In addition, mites with a mixed genetic composition and individual hosts with coinfections (that is, presenting two different strains of mites) were detected. These results are suggestive of multiple introduction events of *S. scabiei* (Dutech et al., 2010; Jezic et al., 2012). Mite variants may be potentially relevant for vicuña conservation, as they could be linked to variations in the severity of clinical outcomes at both the individual and population levels. These associations, along with factors such as ambient conditions, host susceptibility, and food availability, could influence the overall health and survival of vicuñas.

The observed genetic diversity of *S. scabiei* in the Puna is likely related to the ethological characteristics of the host as well as management practices in the region. It is well established that the main mode of transmission for mange is direct contact, which, for wild vicuñas, occurs primarily during breeding, mother‒offspring interactions and antagonist male encounters (Arzamendia et al., 2012; Ferreyra et al., 2022). Furthermore, vicuñas live in families and groups, so shared areas represent a source of indirect contact that could increase transmission opportunities. In addition, the management of wild vicuñas during chakus implies the random capture of free-ranging animals, which increases direct and indirect contact events and might promote contact among animals that would not occur otherwise (Castillo, 2018). In this context, understanding the presence and distribution of sarcoptic mange could help refine capture sites and incorporate specific management practices to minimize the spread of the disease or of a particular strain during this important socioeconomic and sustainable practice. A further characterization of mite variants and their drug susceptibility would be necessary to detect if a strain is, for example, resistant to ivermectin, as has already been observed for other SAC (Galvan et al., 2012), and plan strategies that avoid its spread.

Another relevant factor for the Puna region is the sympatry of vicuñas and domestic livestock, especially llamas, which often seen graze together. For livestock, the trade and exchange of animals among breeders is quite a common practice, including trading with neighbouring Bolivia (Bustamante et al., 2006). With limited targeted surveillance, the chances of introducing multiple *S. scabiei* variants in northern Argentina are far from negligible. Transmission between domestic and wild animals has been previously reported (Matsuyama et al., 2015; Peltier et al., 2017; Fraser et al., 2019), and our results confirmed that domestic llamas and wild vicuñas can share the same mite variants.

In contrast to Jujuy, mites from San Juan and La Rioja were in a single genetic cluster with lower genetic diversity and a greater proportion of fixed alleles than Jujuy (see Table 2). In addition, since SG1 and SG2 consisted of sampling events separated by a 5-year period and LB1 and LB2 by a 4-year period, the single-cluster result appears consistent with a single introduction of one of the mite strains, its persistence over time, and its ongoing expansion across the region. The fact that LB2 includes one sample from Famatina, which is located in a different mountain range than Laguna Brava distant ∼150 km, further supports mange expansion. Adding this information to the timeline of mange reports, our results support the hypothesis of mange spreading from San Guillermo National Park to Laguna Brava Reserve and surrounding areas proposed by Ferreyra et al. (2022). Finally, the results of different sampling events in SG provide further evidence that mange transmission mechanisms are independent of camelid population density (Perrig and Gregorio, 2024), since mites with the same genetic characteristics persist through the dramatic population bottleneck suffered by vicuñas and guanacos in that protected area.

With respect to the currently undetermined origin of the SG outbreak, the microsatellite results revealed a possible connection between the San Juan-La Rioja and Jujuy clusters. Interestingly, some of the mites affecting vicuñas from CY and LF presented mixed components between those groups, and a single mite from CY was rather similar to those collected in the San Juan-La Rioja study area. Although we did not find the SG variant among mites obtained from llamas (all sampled from the Puna of Jujuy), the results show that llamas and vicuñas in Jujuy actually share mite strains. It is therefore our opinion that further efforts should be dedicated to exploring in depth the genetic composition of *Sarcoptes* mites circulating among domestic SAC in the northernmost region of Argentina. The results based on a larger sampling area would allow to robustly test the hypothesis that the SG outbreak originated from the incautious introduction of infested llamas from Jujuy in the context government livestock-incentives program (Ferreyra et al., 2022). An alternative hypothesis is that the SG variant was already circulating among neighbouring populations of wild camelids in Chile and eventually infected the naïve SG counterparts by wildlife-to-wildlife spontaneous contact. Unfortunately, there are no available genetic studies of mangy camelids from the Chilean vicinity of SG; however, reports of wild camelids with abnormal alopecia compatible with sarcoptic mange were registered in protected areas of north-central Chile from 2009 to 2018, but mainly from 2014 to 2018 (Montecino-Latorre et al., 2020). Guanacos are known to exhibit migratory movements, are capable of traveling wide distances (Puig et al., 2011; Carmanchahi et al., 2015), and there is also evidence that they cross the Andes (R. Ovejero, personal communication). Hence that it is important to plan transboundary conservation schemes that include measures to control mange expansion.

This study is one of the few to address the genetic aspects of sarcoptic mange among wild and domestic camelids in South America. In addition to the two study areas, there are other regions in South America where SAC are affected by sarcoptic mange and may pose a risk for other cohabiting species. New outbreaks of unpredictable demographic impact might occur in the future, representing a poorly recognized conservation threat. We have provided new sequences and a baseline for analysing *S. scabiei* inter- and intraspecific transmission patterns and for understanding mite evolution and phylogenetics. This information can also be relevant to design intersectoral strategies for the prevention and control of mange, contributing to the conservation of guanacos and vicuñas and to the maintenance of livestock farming in high-altitude regions.

## Supporting information

Anello et al. supplementary material Anello et al. supplementary material

## Data Availability

The *cox1* sequences generated during the current study are available at GenBank repository under accession numbers PQ137498-PQ137524 (Table S2; https://www.ncbi.nlm.nih.gov/genbank), while the genotypes obtained from microsatellites markers and analysed in this study are available from the corresponding author on reasonable request.

## Supplementary material

The supplementary material for this article can be found at https://doi.org/10.1017/S0031182025000344.
