# Supplementary material for: Molecular analysis of Sarcoptes scabiei infecting wild and domestic South American camelids in Argentina
Source: Parasitology. 2025 Mar 28;152(4):409–18. doi: 10.1017/S0031182025000344 (PMC12186565; doi:10.1017/S0031182025000344)
Supplement: Anello et al. supplementary material [file S0031182025000344sup001.docx]

# **Supplementary materials**

Molecular analysis of *Sarcoptes scabiei* infecting wild and domestic South American camelids in Argentina

Melina Anello^1^, Fabiana Sosa^2,3^, Hebe Ferreyra^4,5^, Rebeca Lobo Allende^6^, Mariana Mastromatey^6^, Marcela Uhart^7^, Sandra Romero^2^, Mónica Florin-Christensen^3,8^, Barbara Moroni^9^, Anna Rita Molinar^10^, Luca Rossi^10^, Florencia Di Rocco^1^

^1^Instituto Multidisciplinario de Biología Celular, CONICET-UNLP-CIC, La Plata, Buenos Aires, Argentina; ^2^ Instituto de Investigación y Desarrollo Tecnológico para la Agricultura Familiar, Región NOA, INTA, Argentina; ^3^Consejo Nacional de Investigaciones Científicas Y Técnicas (CONICET), Argentina; ^4^Dirección Nacional de Conservación, Delegación Regional Centro de la Administración de Parques Nacionales, Argentina; ^5^Universidad Nacional de Villa María, Córdoba, Argentina; ^6^Universidad Nacional de Chilecito, La Rioja, Argentina; ^7^Latin America Program, Karen C. Drayer Wildlife Health Center, School of Veterinary Medicine, University of California, Davis, USA; ^8^Instituto de Patobiología Veterinaria, INTA-CONICET, Centro de Investigaciones en Ciencias Veterinarias y Agronómicas, INTA, Argentina; ^9^Istituto Zooprofilattico Sperimentale di Piemonte, Liguria e Valle d’Aosta, Torino, Italy; ^10^Departamento de Ciencias Veterinarias, Universidad de Torino, Grugliasco, Italy.

**Corresponding author:** Melina Anello, Email: melianello@gmail.com

**Table S1**. Primers used for PCR amplification of *cox1* gene from *S. sacbiei* isolates

| **Primer name** | **Primer sequence (5´-3´)** | **T annealing (°C)** | **Product length (bp)** |
| --- | --- | --- | --- |
| COXI-F | TCATAAAACGACCTTTAACTTAGCA | 62 | 1959 |
| COXI-R | AAGATCAATATCATTGATGAGCCAC |  |  |
| COXIfrag1-F | AGATGTTGATACTCGAGCTTATTT | 54 | 619 |
| COXIfrag1-R | GTGAATTTCCCTAGGATAGGAGT |  |  |
| COXIfrag2-F | TGGAACTGGTAGAGGAACTG | 54 | 573 |
| COXIfrag2-R | GTAGCTGAAGTAAAATAAGCTCG |  |  |

**Table S2.** *S. scabiei* isolates analysed for *cox1* gene sequences in this study. Included data are sample ID, geographical origin, host species, and GenBank accession number.

| **Sample ID** | **Origin** | **Host** | **GenBank accession number for COXI sequence** |
| --- | --- | --- | --- |
| Sarcoptes_scabiei_isolate_LFV1.1 | Argentina: Lagunillas del Farallon, Jujuy | *Vicugna vicugna* | PQ137498 |
| Sarcoptes_scabiei_isolate_LFV2.1 | Argentina: Lagunillas del Farallon, Jujuy | *Vicugna vicugna* | PQ137499 |
| Sarcoptes_scabiei_isolate_LFV3.1 | Argentina: Lagunillas del Farallon, Jujuy | *Vicugna vicugna* | PQ137500 |
| Sarcoptes_scabiei_isolate_LFV4.1 | Argentina: Lagunillas del Farallon, Jujuy | *Vicugna vicugna* | PQ137501 |
| Sarcoptes_scabiei_isolate_LFV6.1 | Argentina: Lagunillas del Farallon, Jujuy | *Vicugna vicugna* | PQ137502 |
| Sarcoptes_scabiei_isolate_SG1VM5 | Argentina: Parque Nacional San Guillermo, San Juan | *Vicugna vicugna* | PQ137503 |
| Sarcoptes_scabiei_isolate_SG1VM4 | Argentina: Parque Nacional San Guillermo, San Juan | *Vicugna vicugna* | PQ137504 |
| Sarcoptes_scabiei_isolate_SG1VM6 | Argentina: Parque Nacional San Guillermo, San Juan | *Vicugna vicugna* | PQ137505 |
| Sarcoptes_scabiei_isolate_SG1GM1 | Argentina: Parque Nacional San Guillermo, San Juan | *Lama guanicoe* | PQ137506 |
| Sarcoptes_scabiei_isolate_LB1GM2 | Argentina: Reserva Provincial Laguna Brava, La Rioja | *Lama guanicoe* | PQ137507 |
| Sarcoptes_scabiei_isolate_LB1GM3 | Argentina: Reserva Provincial Laguna Brava, La Rioja | *Lama guanicoe* | PQ137508 |
| Sarcoptes_scabiei_isolate_SG1GM9 | Argentina: Parque Nacional San Guillermo, San Juan | *Lama guanicoe* | PQ137509 |
| Sarcoptes_scabiei_isolate_CNLL1P | Argentina: Cieneguillas, Jujuy | *Lama glama* | PQ137510 |
| Sarcoptes_scabiei_isolate_CNLL2pam | Argentina: Cieneguillas, Jujuy | *Lama glama* | PQ137511 |
| Sarcoptes_scabiei_isolate_CNLL3i | Argentina: Cieneguillas, Jujuy | *Lama glama* | PQ137512 |
| Sarcoptes_scabiei_isolate_LB1VM1 | Argentina: Reserva Provincial Laguna Brava, La Rioja | *Vicugna vicugna* | PQ137513 |
| Sarcoptes_scabiei_isolate_SG1VM15 | Argentina: Parque Nacional San Guillermo, San Juan | *Vicugna vicugna* | PQ137514 |
| Sarcoptes_scabiei_isolate_SG1VM20 | Argentina: Parque Nacional San Guillermo, San Juan | *Vicugna vicugna* | PQ137515 |
| Sarcoptes_scabiei_isolate_SG1VM131 | Argentina: Parque Nacional San Guillermo, San Juan | *Vicugna vicugna* | PQ137516 |
| Sarcoptes_scabiei_isolate_SG2GRB | Argentina: Rodeo, San Juan | *Lama guanicoe* | PQ137517 |
| Sarcoptes_scabiei_isolate_CYV28A | Argentina: Coyaguayma, Jujuy | *Vicugna vicugna* | PQ137518 |
| Sarcoptes_scabiei_isolate_LFV13B | Argentina: Lagunillas del Farallon, Jujuy | *Vicugna vicugna* | PQ137519 |
| Sarcoptes_scabiei_isolate_SG2VMAJB | Argentina: Reserva Provincial San Guillermo, San Juan | *Vicugna vicugna* | PQ137520 |
| Sarcoptes_scabiei_isolate_QUV1A | Argentina: Quera, Jujuy | *Vicugna vicugna* | PQ137521 |
| Sarcoptes_scabiei_isolate_QUV24A | Argentina: Quera, Jujuy | *Vicugna vicugna* | PQ137522 |
| Sarcoptes_scabiei_isolate_SG2V2A | Argentina: Reserva Provincial San Guillermo, San Juan | *Vicugna vicugna* | PQ137523 |
| Sarcoptes_scabiei_isolate_LFV38B | Argentina: Lagunillas del Farallon, Jujuy | *Vicugna vicugna* | PQ137524 |

**
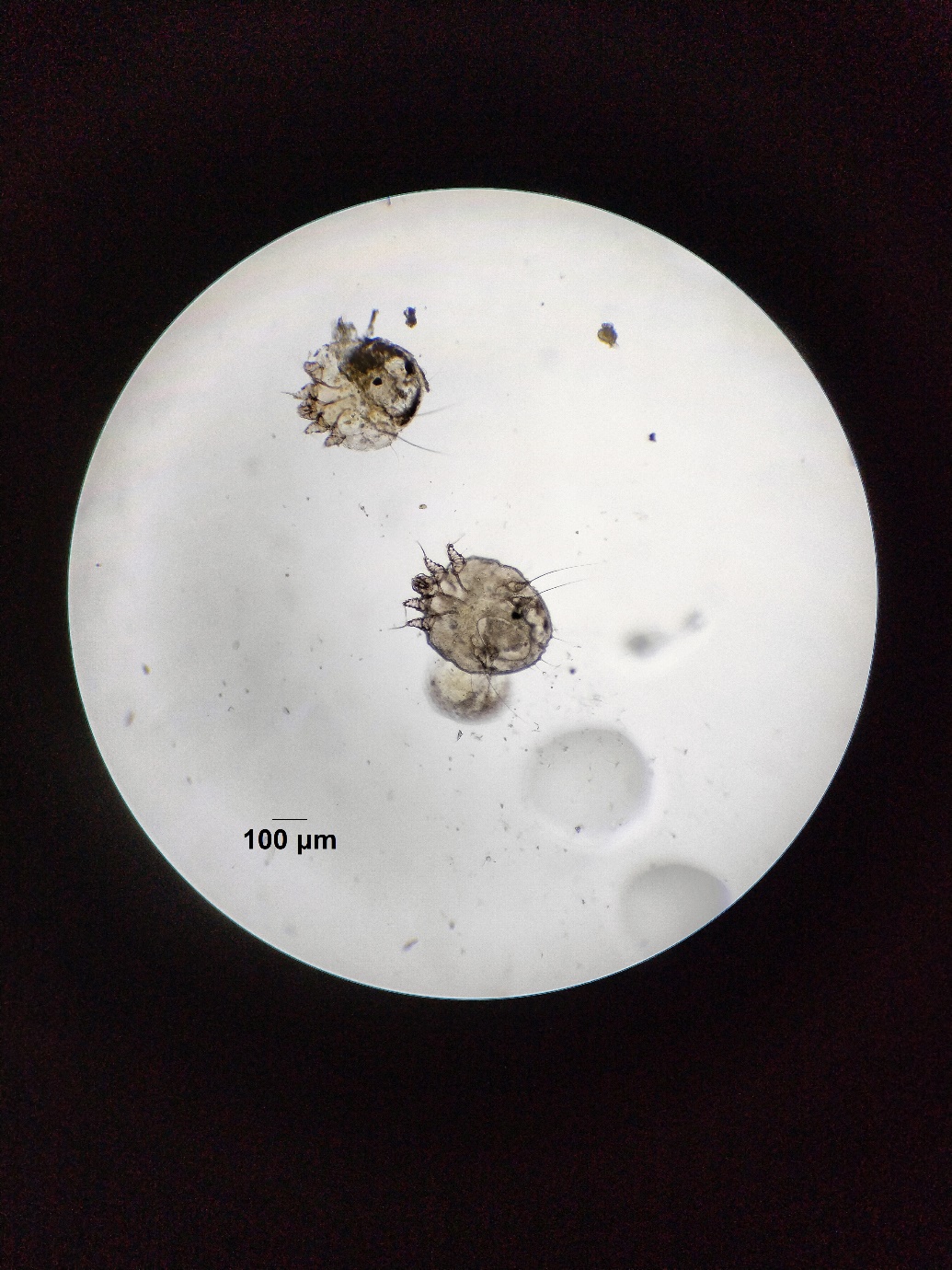
**

**Figure S1.** Microscopic photograph of two representative mites isolated from SACs included in this study.


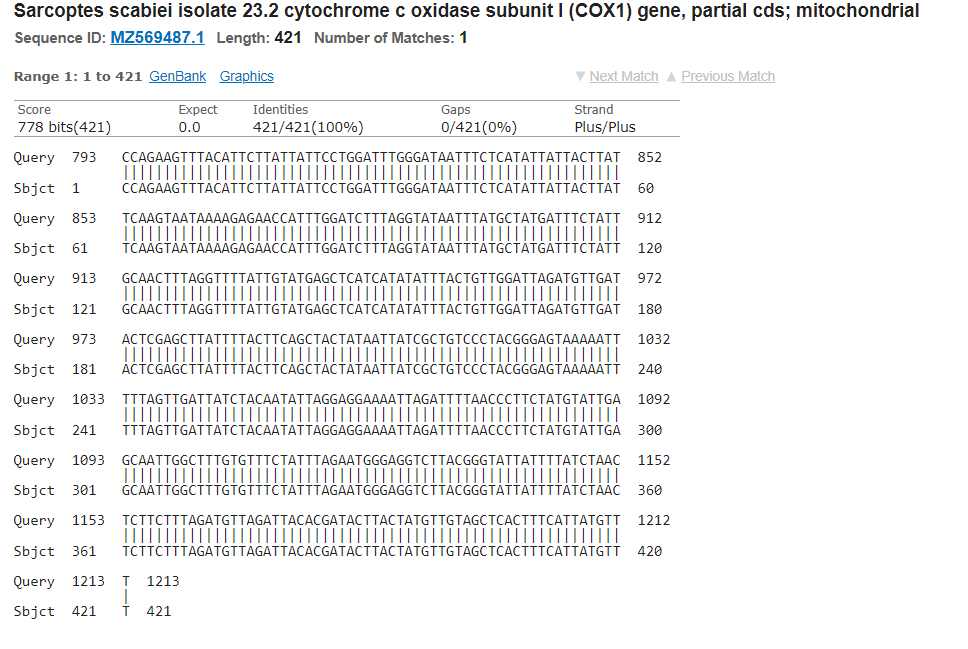


A


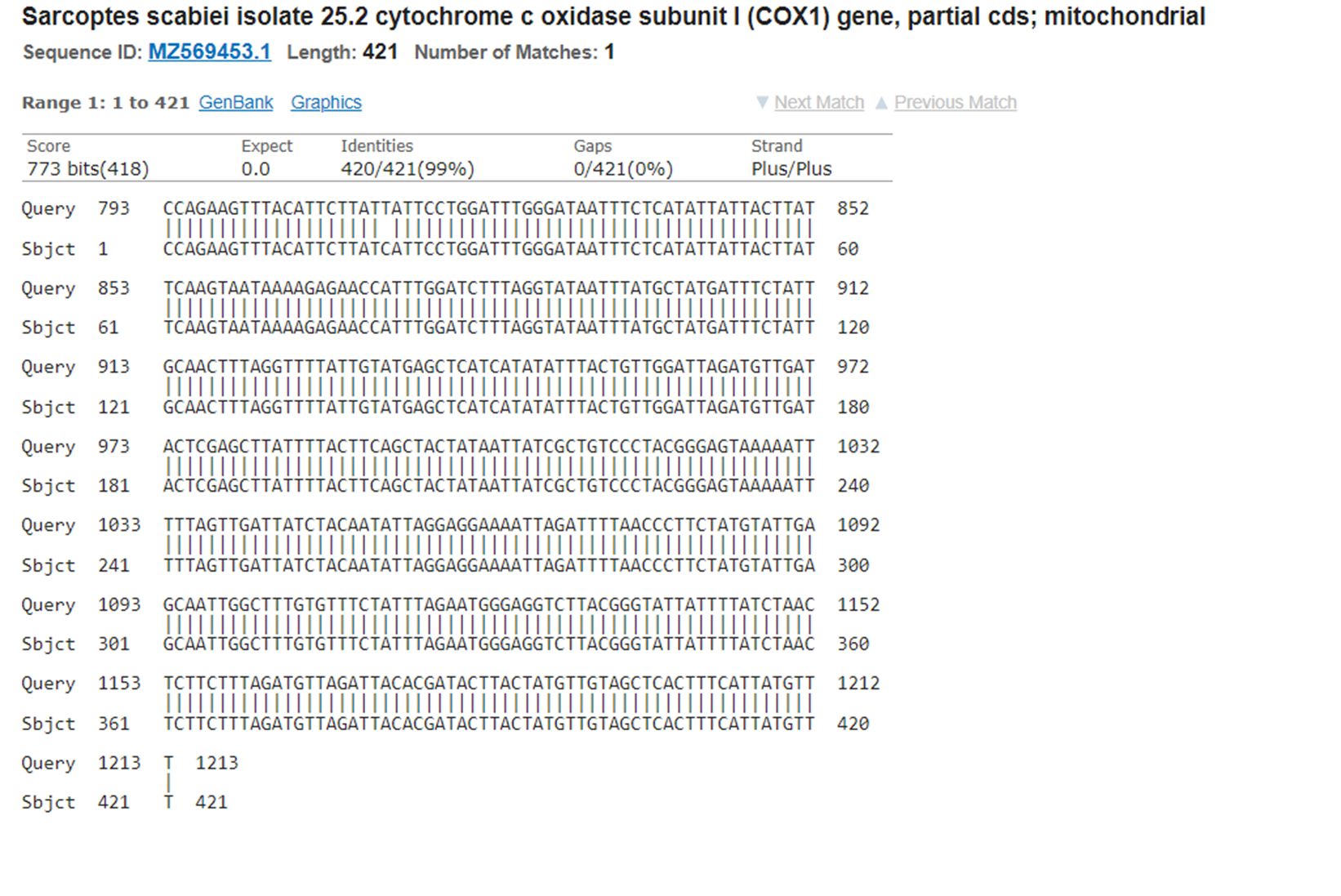


B


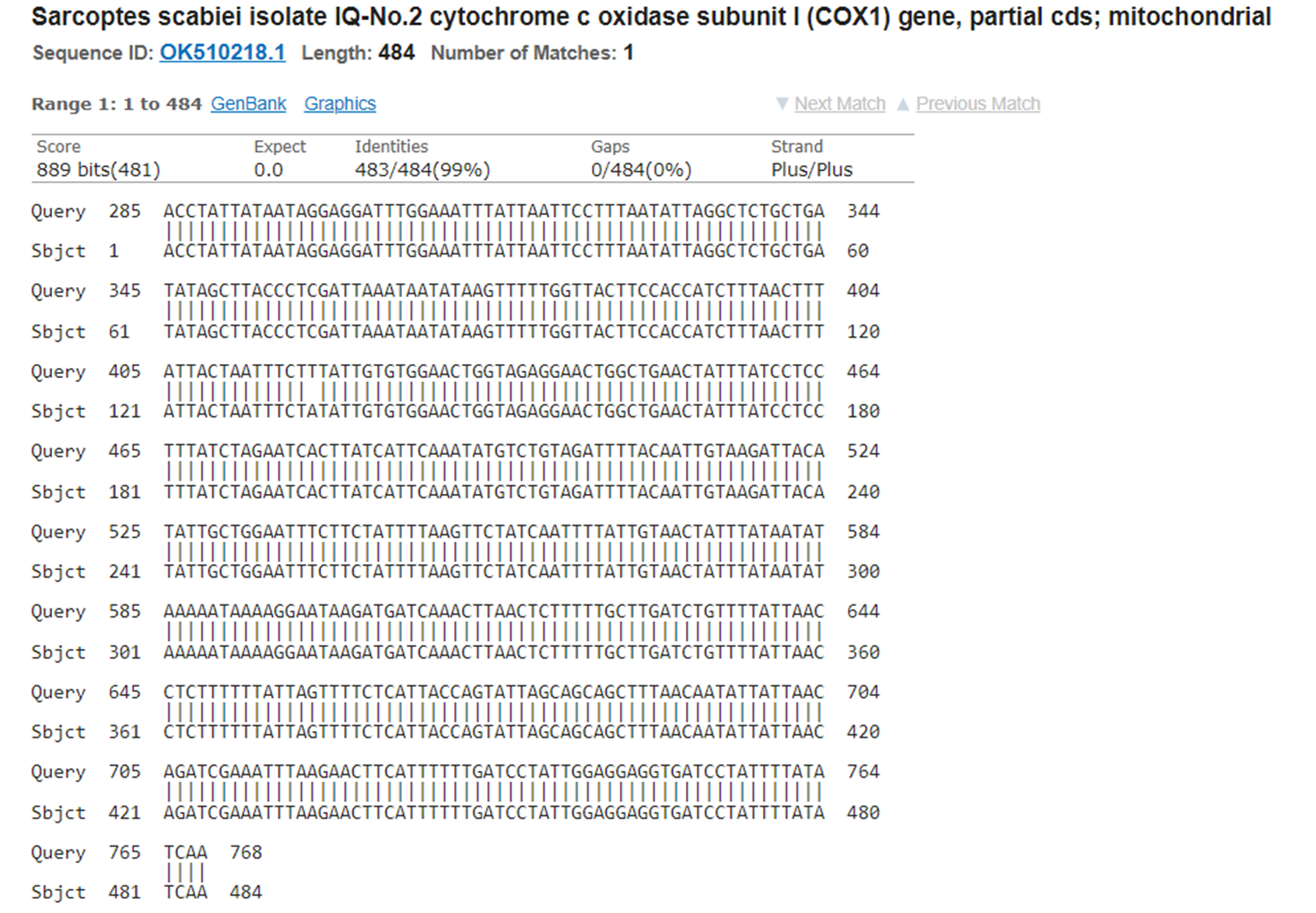


C

**Figure S2**. *cox1* pairwise alignment between the haplotype obtained from Argentinian samples (referred to as “Query”) and: two distinct fragments of 421 bp isolated from vicuñas in the southern Peruvian Andes (as reported by Gomez-Puerta *et al*., 2022) (A and B); a 484 bp fragment isolated from camels in Iraq (as documented by Al-Hasnawy *et al*., 2022).


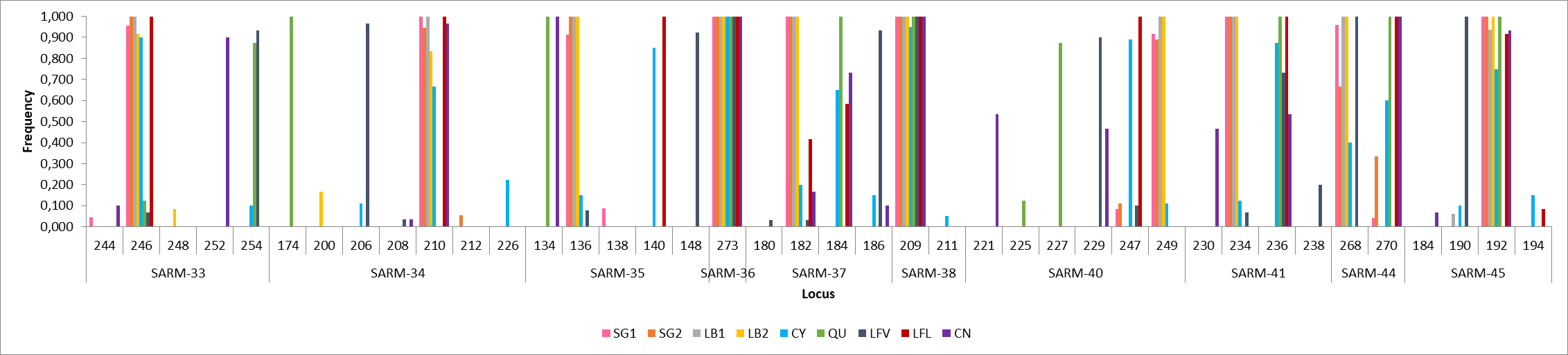


**Figure S3**. Plot of allele frequency obtained for each of the 10 microsatellite markers analysed from *S. scabiei* mites.


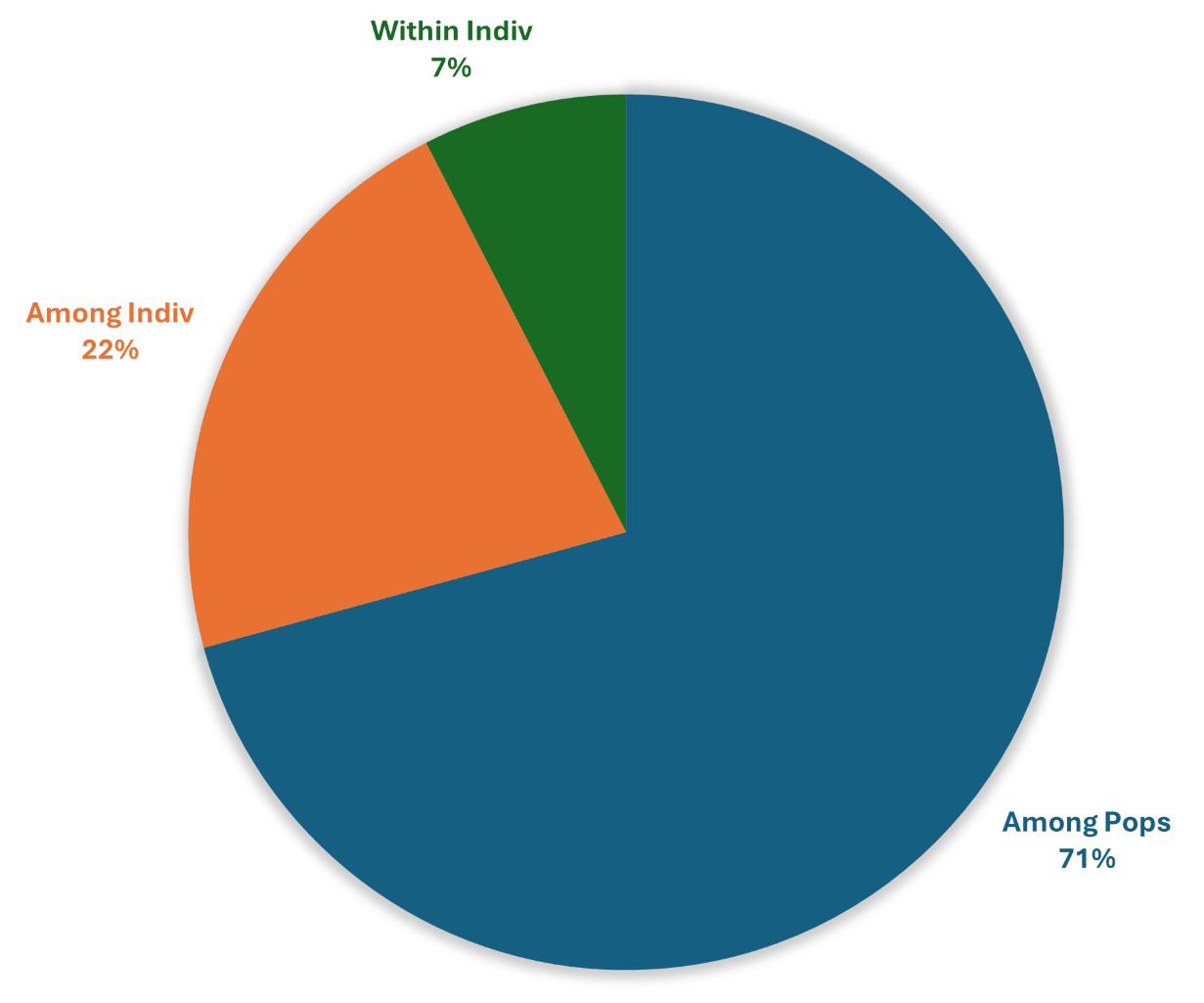


**Figure S4**. Circle plot illustrating the AMOVA results for genetic differentiation obtained from the analysis of 10 microsatellite markers in *S. scabiei* mites.


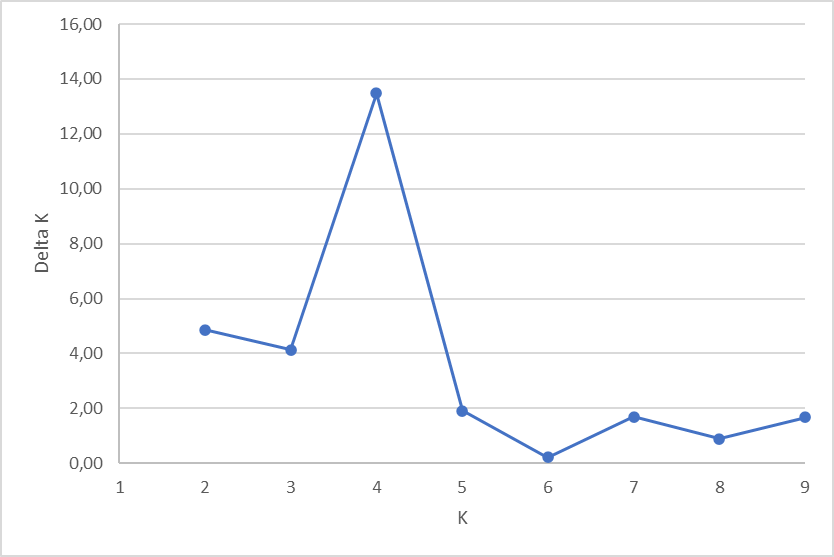

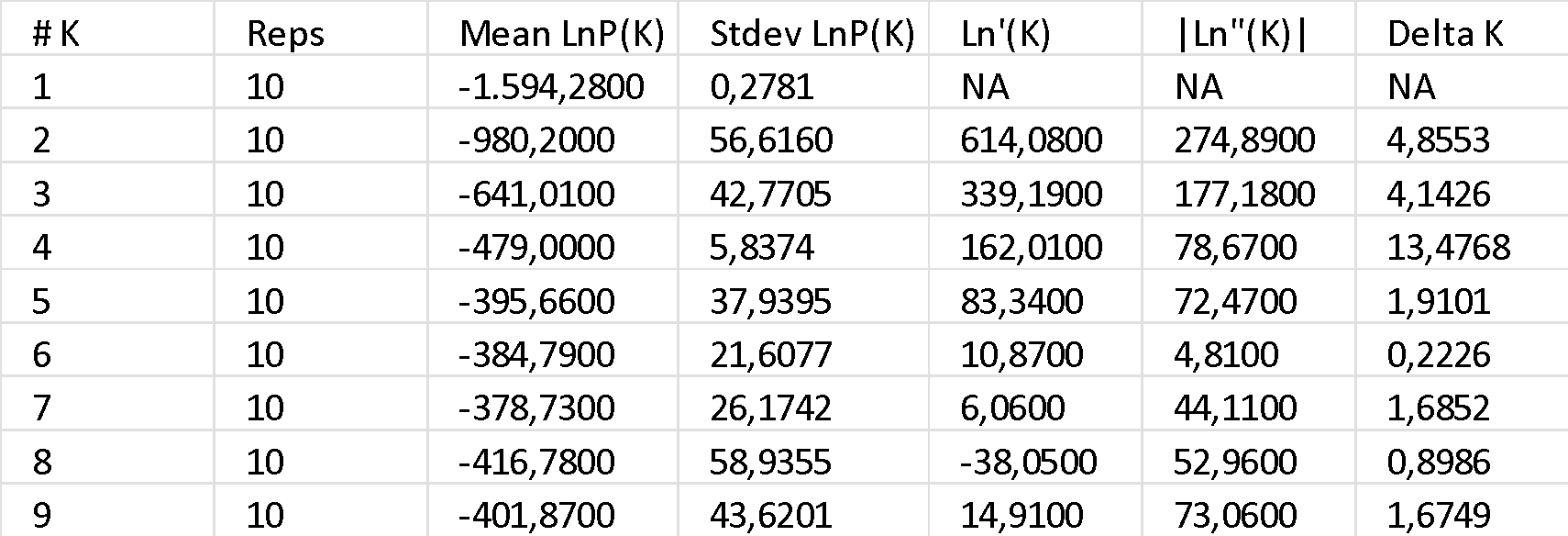


A

B

**Figure S5.** Evanno method results for genetic differentiation based on analysis of 10 microsatellite markers in *S. scabiei* mites. Panel A shows the plot of Delta K versus K, while panel B presents a table listing the parameters for K-values ranging from 1 to 9.


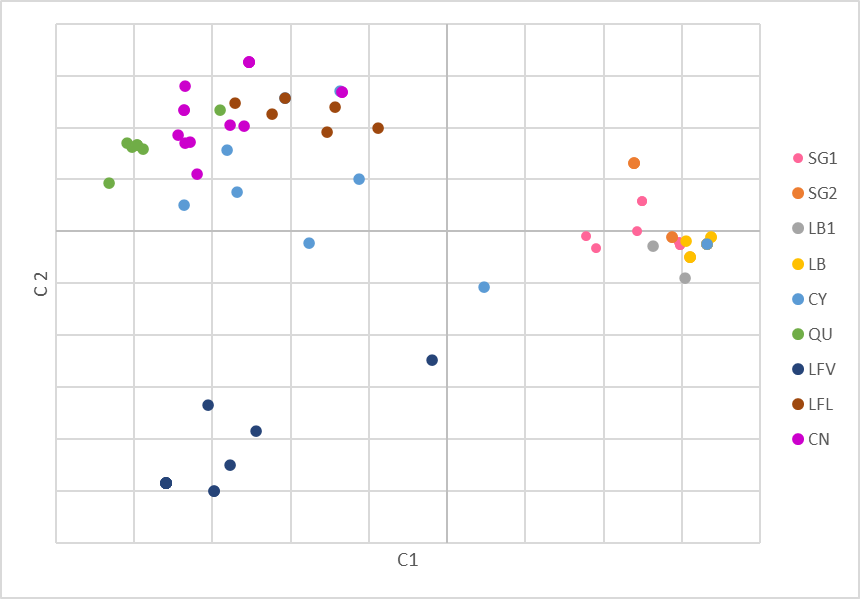


A


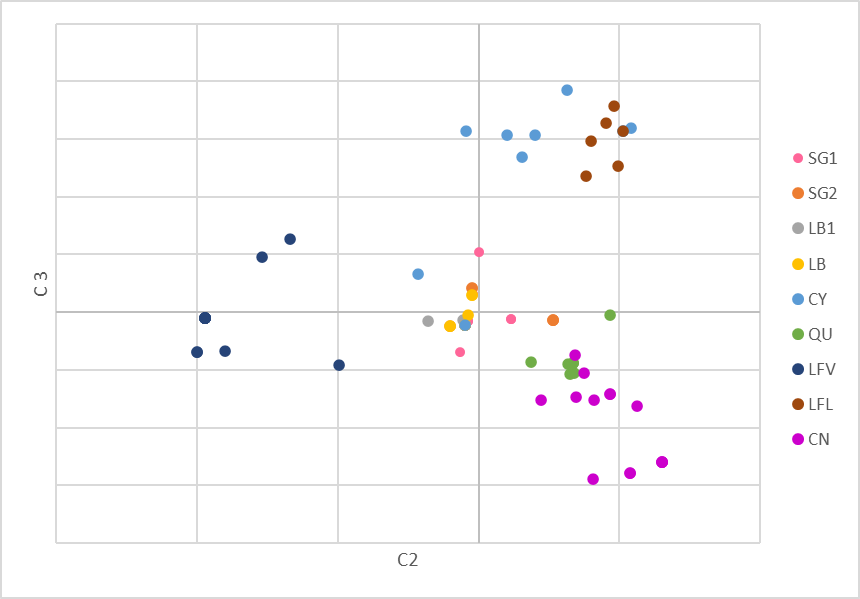


B


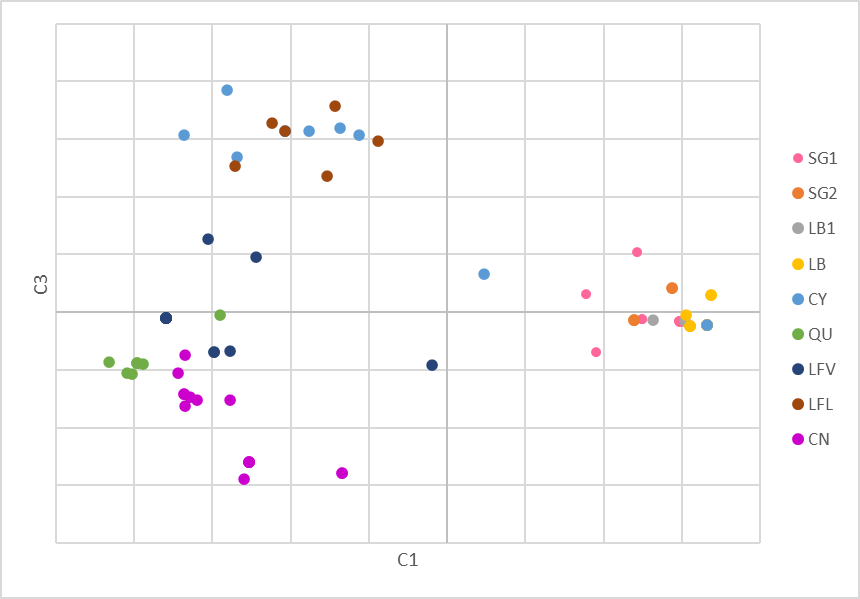


C

**Figure S6**. MDS plots for components 1 vs. 2 (A), 2 vs. 3 (B) and 1 vs. 3. Each colour-coded dot represents a single mite from the corresponding sampling site, as indicated by the reference panel at the right of the plots.
